# Urinary Levels of Cathepsin B in Preterm Newborns

**DOI:** 10.3390/jcm10184254

**Published:** 2021-09-19

**Authors:** Monika Kamianowska, Marek Szczepański, Anna Krukowska, Aleksandra Kamianowska, Anna Wasilewska

**Affiliations:** 1 Department of Neonatology and Neonatal Intensive Care, Medical University of Bialystok, 15-276 Białystok, Poland; szczepanski5@gazeta.pl (M.S.); anna_krukowska1@wp.pl (A.K.); 2 Department of Pediatrics and Nephrology, Medical University of Bialystok, 15-276 Białystok, Poland; olcikkam@wp.pl (A.K.); annwasil@interia.pl (A.W.)

**Keywords:** cathepsin B, tubular damage, premature neonates, immaturity

## Abstract

Increased investment in perinatal health in developing countries has improved the survival of preterm newborns, but their significant multiorgan immaturity is associated with short and long-term adverse consequences. Cathepsin B, as a protease with angiogenic properties, may be related to the process of nephrogenesis. A total of 88 neonates (60 premature children, 28 healthy term children) were included in this prospective study. We collected urine samples on the first or second day of life. In order to determine the concentration of cathepsin B in the urine, the commercially available enzyme immunoassay was used. The urinary concentrations of cathepsin B normalized with the urinary concentrations of creatinine (cathepsin B/Cr.) in newborns born at 30–34, 35–36, and 37–41 (the control group) weeks of pregnancy were (median, Q1–Q3) 4.00 (2.82–5.12), 3.07 (1.95–3.90), and 2.51 (2.00–3.48) ng/mg Cr, respectively. Statistically significant differences were found between the group of newborns born at 30–34 weeks of pregnancy and the control group (*p* < 0.01), and between early and late preterm babies (PTB) (*p* < 0.05). The group of children born at 35–36 weeks of pregnancy and the control group did not differ significantly. This result suggests that the elevated urinary cathepsin B/Cr. level may be the result of the kidneys’ immaturity in preterm newborns.

## 1. Introduction

Preterm birth (before 37 completed weeks of gestation) accounts for 11% of births worldwide [1]. Increased investment in perinatal health in developing countries and interventions such as antenatal steroids have improved survival in this group of children, but significant multiorgan immaturity is associated with short and long-term adverse consequences [2]. The first kidney glomeruli form at 9–10 weeks of gestation [3]. During the late second and third trimester, there is an exponential increase in the number of nephrons between 18 and 32 weeks [4,5]. Nephrogenesis in humans ends by approximately 34–36 weeks of gestation, with over 60% of nephrons being formed during the last trimester [6]. Hence, in premature neonates, normal nephrogenesis is interrupted, and both nephron number and kidney size are reduced [7]. While nephrogenesis may continue in premature neonates for up to 40 days following birth, these nephrons are not normal and age at an increased rate [8]. However, despite this postnatal development of the kidneys, premature children are still left with a lower number of nephrons. For example, a premature neonate born at 26 weeks of gestation, despite 40 additional days of nephrogenesis, will only have nephron development until 32 weeks as opposed to continuing nephrogenesis to 36 weeks in term gestation [9,10]. Kidney injury from hypoperfusion and drug nephrotoxicity leads to further frequently unnoticed changes in kidney structure and function. Thus, the glomerular and tubular maturation of the kidneys of preterm newborns may be confounded by the nephropathy of prematurity and acute kidney injury (AKI), whose incidence in neonates is estimated at 8–24% of children hospitalised in the Intensive Neonatal Care Units. One-third of this group are premature babies [7,11,12,13].

Cathepsin B is a lysosomal cysteine protease synthesized on the rough endoplasmic reticulum as a pre-proenzyme; cathepsin B in its mature, double-chain form comprises a heavy chain and a light chain [14,15,16,17]. Cathepsin B is a protein belonging to hydrolases and is involved in the processing of hormones and proteins, regulation of the cell cycle, autophagy, and cell death [18,19]. In kidneys, cathepsin B is found in proximal tubule cells. It is involved in the digestion of proteins reabsorbed from the tubular fluid following glomerular filtration. A small amount of this protease can be detected in urine under physiological conditions. Its levels increase because of tubular damage or renal dysfunction [18,20]. During pregnancy, cathepsin B is predominantly found in placental and decidual macrophages, which may be important in the mediation of villous angiogenesis and decidual apoptosis [21]. With tumours, the expression of cathepsin B correlates with angiogenesis and is thought to promote the remodeling of the extracellular matrix to permit neovascularization [22,23].

This study assessed whether cathepsin B may be involved in the maturation of the foetus’ kidneys by evaluating the effect of prematurity on the concentration of cathepsin B in the urine of preterm newborns.

## 2. Patients and Methods

### 2.1. Patient Recruitment and Sample Collection

A total of 88 neonates were included in this prospective study. Sixty of them were born prematurely at 30–36 weeks of pregnancy. The neonates were hospitalized in the Department of Neonatology and Intensive Neonatal Care at the Medical University of Bialystok, Poland between December 2017 and December 2018. The study was conducted according to the guidelines of the Declaration of Helsinki, and approved by the Local Bioethics Committee of The Medical University of Bialystok (protocol code: R-I-002/360/2016, date of approval: 17 October 2016). Prior to the study written informed consent has been obtained from the parents of all the neonates. The clinical condition of the neonates was good or average; they were appropriate for gestational age (AGA), with the weight between the 10th and 90th centile of birth weight for their gestational age using normalized growth curves [24]. The group of premature babies born at 30–36 weeks of pregnancy were divided into two subgroups: 28 children born between 30 and 34 weeks of pregnancy (hospitalized in the ward for preterm babies) and 32 children born between 35 and 36 weeks of pregnancy (hospitalized in a rooming-in ward). The reference group comprised 28 healthy babies. These neonates developed through normal pregnancies, with no prenatal and perinatal complications.

The inclusion criteria for this study were normal prenatal and postnatal ultrasound examination results of the kidneys, and a good or average clinical condition. The exclusion criteria were: 1-min Apgar scores < 4; congenital abnormalities, including urinary tract defects (polycystic kidney disease, hydronephrosis, duplex kidneys/ureters, renal agenesis, or other anatomical abnormalities); inborn error of metabolism; heart disease; abnormal ultrasound examination of the kidneys and the central nervous system (hyper-echoic zones around the lateral ventricles of the brain and intraventricular hemorrhage grade I and II were accepted); abnormal laboratory tests (including elevated levels of inflammatory markers); treatment with catecholamines, antibiotics, diuretics, or mechanical ventilation. Additionally, the children of mothers with a burdened medical history were excluded from the study.

Collection of urine samples in the study group was performed using single-use sterile bags (Medres, Zabrze, Poland). Urine was collected once on the first or second day of life. The urine samples obtained after centrifugation were kept in the refrigerator (4 °C) for no longer than 2 h and then frozen at −80 °C. Repeated freeze–thaw cycles were not used. Collection of the blood samples was conducted during the first or second day of the neonate’s life during routine practice in the Unit. S-Monovette 1.2 mL, Clotting Activator/Serum test tubes (Sarstedt AG & Co., Nümbrecht, Germany) for venous blood sampling were used. A blood cell morphology test and blood biochemistry tests were performed right after taking the blood samples.

### 2.2. Determination of Urinary Cathepsin

The levels of urinary cathepsin B were determined using a commercially available ELISA kit (Cloud–Clone Corp., Katy, TX, USA), according to manufacturer’s instructions, and were expressed in nanograms per milliliter. The detection range was 0.312–20 ng/mL, according to specifications of the kit. The mean intra-assay and inter-assay coefficients of variation (CV) for cathepsin B were <10% and <12%, respectively.

In order to eliminate the potential confounding effect of urinary dilution, we normalized the urinary cathepsin B concentrations for the urinary concentration of creatinine; this was expressed in nanograms per milligram of creatinine (ng/mg Cr). The urinary concentration of creatinine was determined using Jaffé’s method.

Calculation of estimated GFR was performed using the Schwartz formula (for the preterm babies, eGFR = 0.33 × L/Scr.; for term babies, eGFR = 0.45 × L/Scr., where L is the length in centimeters and Scr. is serum creatinine in milligrams per deciliter).

The determination of urinary cathepsin B was performed in the Department’s Laboratory of Pediatrics and Nephrology, at the Medical University of Bialystok. The morphology and serum biochemistry tests were performed in the Department of Laboratory Diagnostics at the University Clinical Hospital in Bialystok.

### 2.3. Statistical Analysis

The statistical analysis was completed using Statistica 13.3 package (StatSoft, Cracow, Poland). It expressed discrete variables as counts (percentage), continuous variables as median and quartiles (Q1–Q3). In order to determine normal distribution, the Shapiro–Wilk test was used. The Mann–Whitney U test was used for intergroup comparisons of continuous variables because the data were not normally distributed. To establish the direction and power of association between urinary cathepsin B concentrations and other variables, Spearman’s rank correlation coefficients were used. The results were significant at *p* < 0.05.

## 3. Results

Eighty-eight neonates were included in the study. Twenty-eight of them were healthy neonates, and sixty were born prematurely at 30–36 weeks of pregnancy. Both groups were sex-matched (*p* > 0.05). Birth weight, length, and head and chest circuits were significantly lower in premature neonates than in term children; however, all the children were appropriate for gestational age. Table 1 presents the characteristics of premature babies.

In both examined groups of premature babies (born at 30–34 and at 35–36 weeks of pregnancy), there were no statistically significant differences in the type of delivery (vaginal delivery or caesarean delivery). Both groups were sex-matched (*p* > 0.05). Birth weight was statistically significantly lower in neonates born at 30–34 weeks of pregnancy than in babies born at 35–36 weeks of pregnancy. All premature babies were appropriate for gestational age, and when we divided both groups of preterm neonates according to the percentile of birth weight (10–50 percentile and 51–90 percentile), they were matched according to their percentile of birth weight. Both 5-min and 10-min Apgar scores were ≥8 in all neonates, and lower 1-min and 3-min Apgar scores characterized younger children.

In both groups of preterm neonates, blood morphology and biochemical tests were normal. Statistically significantly higher number of leucocytes, urea, and alanine aminotransferase concentrations were found in younger children (Table 2).

All neonates had normal renal function parameters (serum and urinary concentration of creatinine, estimated GFR, and urine output). The serum concentration of creatinine did not show a statistically significant difference between the groups. The serum concentration of creatinine was higher in neonates born by cesarean delivery when compared to children born by vaginal delivery. The difference was close to being statistically significant (*p* = 0.053). Urinary creatinine was significantly lower in premature babies when compared to the reference group (*p* < 0.01). The lowest concentration of urinary creatinine was found in children born at 30–34 weeks. Similarly, eGFR was significantly lower in premature children compared to healthy controls (*p* < 0.01); however, we did not notice any difference in eGFR between children born at 30–34 weeks and 35–36 weeks (*p* > 0.05) (Table 3).

In the entire group of premature neonates, the urinary level of cathepsin B/Cr. was higher compared to the reference group (*p* < 0.05). The highest urinary excretion of cathepsin B/Cr. was found in babies born at 30–34 weeks of pregnancy, and the difference was statistically significant when compared to the reference group (*p* < 0.01) and when compared to babies born at 35–36 weeks.(*p* < 0.05). However, no statistically significant difference in the urinary level of cathepsin B/Cr. was observed between children born at 35–36 weeks of pregnancy and the reference group.

The analysis did not show any relationship between the concentration of urinary cathepsin B/Cr. and the way of delivery (vaginal delivery or caesarean delivery), Apgar score, prenatal steroid therapy, use of parenteral nutrition, use of nCPAP, or oxygen therapy. No statistically significant differences in the concentrations of urinary cathepsin B/Cr. were found between the groups of boys and girls.

## 4. Discussion

Kidney immaturity as a consequence of preterm delivery and perinatal problems connected to prematurity such as respiratory and circulatory failure, asphyxia, and antibiotic therapy are risk factors of renal tubular injury in premature neonates. The aim of this prospective study was to determine the values of urinary cathepsin B in premature neonates. It was hypothesized that cathepsin B may be involved in the processes of kidney maturation in the foetus.

Normally, cathepsin B is highly expressed in the proximal tubule. It is the main enzyme involved in the lysosomal digestion of proteins, which are reabsorbed via endocytosis from the glomerular ultrafiltrate. This protease has long been taken under consideration as a biomarker of tubular damage because in response to damages, its levels are lower in tubules and higher in urine [18]. It has recently been shown that the excessive reabsorption of ultra-filtered proteins by proximal tubular cells induces tubular damage and apoptosis/necrosis through the exhaustion of the lysosomal degradation pathway and the leakage of lysosomal enzymes such as cathepsin B into the cytoplasm and urine [20]. The prominent cytoplasmic release of cathepsin B in tubular epithelial cells is crucial for tubular cell injury and the activation of a cytoplasmic macromolecular complex involved in the progression of kidney diseases [25]. Studies conducted on animals showed that cathepsin B is involved in kidney diseases [26,27,28,29]. Wang et al. showed that in human proximal tubular epithelial cells undergoing apoptosis, expression levels and the activation of cathepsin B are increased, and that the serum cathepsin B level was associated with aging-related cardiovascular-renal parameters even in healthy people [30,31,32]. In the study conducted by Piwowar et al., cathepsin B activity increased significantly (*p* < 0.001) in the urine of diabetic patients as compared to the control group, and they concluded that it may be useful as a non-invasive surrogate marker of incipient nephropathy [33].

Knowing that many factors can contribute to tubular damage, we identified a group of sixty children with birth weights appropriate for the gestational age and clinical conditions assessed as good or medium. The children included in this study did not need drugs and mechanical ventilation. Their prenatal and postnatal ultrasound examination of kidneys was normal, and no deviations in laboratory tests and in parameters of renal function were found.

The significantly highest urinary level of cathepsin B/Cr. was observed in neonates born at 30–34 weeks of pregnancy. The lack of statistically significant differences between the urinary level of cathepsin B/Cr. in children born at 35–36 weeks of pregnancy and the reference group was caused by the fact that these neonates were born close to the term of delivery. Thus, it may be supposed that the stages of development of the kidneys in both groups were similar. The significantly highest urinary level of cathepsin B/Cr. was found in neonates born at 30–34 weeks. This may have resulted from the fact that cathepsin B may take part in the process of nephrogenesis as a protease with angiogenic properties [21,22,23,34].

Aisa et al. studied cathepsin B activity in the urine of neonates with intrauterine growth-restricted (IUGR) (median gestational age—36 weeks) and preterm neonates (median gestational age—35 weeks), both at 30–40 days of the corrected age. They found that cathepsin B activity in the urine was statistically significantly increased in preterm children, and even more increased in neonates with IUGR, when compared to the control group (median, (Q1–Q3)): 2.303, (1.7–2.582); 3.633, (2.146–4.848); 1.044, (0.8335–1.372) IU/min mmol, respectively. Taking into consideration significantly lower total renal volume, cortical volume, and observed proteinuria in the IUGR and preterm neonates, they suggested that cathepsin B activity may be useful in the early prediction of renal susceptibility to damage in this group of children [35].

In order to determine if other factors besides immaturity could affect the renal tubules’ functions, the relationship of the concentration of urinary cathepsin B and urinary cathepsin B/Cr. with gender, way of delivery, birth weight, centile of birth weight, Apgar score, prenatal steroid therapy, respiratory disorders (use of nCPAP, oxygen therapy), and the use of parenteral nutrition was examined. However, no correlations were found between them. This result suggests that elevated urinary cathepsin B/Cr. level may be the result of the kidneys’ immaturity in preterm newborns.

The main limitation for this study was the sample size of recruited children, which was quite small, as it was very difficult to get study consent from some parents.

## 5. Conclusions

In conclusion, the results showed that preterm neonates born at 30–34 weeks of gestation had elevated urinary cathepsin B/Cr. levels, which may be a result of the immaturity of kidneys. This preliminary observation should be confirmed in a multicenter study, which would consider not only preterm neonates with birth weights appropriate for gestational age and clinical conditions assessed as good or medium, but also those with clinical conditions assessed as bad, with or without AKI. Certainly, extending the study to the following weeks of life of the studied children, performing them both during hospitalization and after discharge from the hospital, would provide valuable information, confirming the role of cathepsin B as a biomarker of kidney maturity.

## Figures and Tables

**Table 1:** Characteristics of premature babies.

Parameters	Premature			*p*
	(30–36 Weeks) (*n* = 60)	(30–34 Weeks) (*n* = 28)	(35–36 Weeks) (*n* = 32)	
	Median (Q1–Q3)			
Gestational age (weeks)	35 (33–36)	33 (32–34)	36 (35–36)	<0.01
Vaginal delivery/caesarean delivery	16/44	10/22	6/22	NS
Gender (boys/girls)	33/27	16/12	17/15	NS
Birth weight (g)	2450 (2195–2740)	2295 (1720–2450)	2620 (2415–2800)	<0.01
Birth weight (10th–50th percentile/ 51st–90th percentile)	17/43	7/21	10/22	NS
Length (cm)	50.00 (47.00–52.00)	48.00 (45.00–50.00)	52.00 (49.50–53.00)	<0.01
Chest circuit (cm)	30.00 (28.00–31.00)	28.50 (26.50–30.00)	31.00 (30.00–32.00)	<0.01
Head circuit (cm)	32.00 (31.00–33.50)	31.00 (29.00–32.00)	33.00 (32.00–34.00)	<0.01
Prenatal steroid therapy	12	12	0	<0.01
1–min Apgar score (8–10/4–7)	39/21	13/15	26/6	<0.05
3–min Apgar score (8–10/4–7)	47/13	18/10	29/3	<0.05
5–min Apgar score (8–10/4–7)	60/0	28/0	32/0	NS
10–min Apgar score (8–10/4–7)	60/0	28/0	32/0	NS
Oxygen therapy	24	19	5	<0.01
nCPAP	18	17	1	<0.01
Parenteral nutrition	29	24	5	<0.01

*p*—comparison of children born at 30–34 weeks and 35–36 weeks of pregnancy; NS—non statistical; nCPAP—nasal continuous positive airway pressure.

**Table 2:** Basic laboratory results of premature neonates.

Parameters	Premature			*p*
	(30–36 Weeks) (*n* = 60)	(30–34 Weeks) (*n* = 28)	(35–36 Weeks) (*n* = 32)	
Blood morphology				
Leukocytes ×10^3^/µL	14.65 (11.57–17.2)	11.57 (10.04–15.58)	16.53 (14.02–18.92)	0.01
Hemoglobin (g/dL)	17.95 (16.70–18.95)	17.75 (15.60–19.05)	18.11 (16.90–18.95)	NS
Hematocrit (%)	49.95 (46.60–52.51)	49.20 (42.55–52.75)	50.10 (47.40–52.40)	NS
Platelets ×10^3^/µL	258.00 (210.00–293.50)	267.00 (232.00–300.50)	247.50 (204.00–290.50)	NS
Biochemical tests—results				
Urea (mg/dL)	25.50 (18.00–32.00)	29.50 (22.00–45.50)	22.50 (15.00–29.50)	0.01
Serum creatinine (mg/dL)	0.66 (0.61–0.75)	0.64 (0.59–0.68)	0.70 (0.63–0.76)	NS
eGFR (mL/min/1.73 m^2^)	24.58 (21.71–27.11)	24.38 (21.45–27.82)	24.86 (21.99–26.60)	NS
Aspartate aminotransferase (IU/L)	48.50 (38.00–59.50)	40.50 (34.50–59.00)	22.50 (15.00–29.50)	NS
Alanine aminotransferase (IU/L)	10.00 (7.00–13.00)	8.00 (6.00–11.00)	11.50 (9.00–14.50)	0.01
Bilirubin (mg/dL)	5.19 (4.05–6.20)	5.45 (3.90–6.25)	4.80 (4.10–6.13)	NS
Protein (mg/dL)	4.85 (4.40–5.20)	4.85 (4.55–5.20)	4.85 (4.30–5.20)	NS
Sodium (mmol/L)	141.50 (139.00–143.00)	141.00 (138.00–143.00)	141.00 (140.00–143.00)	NS
Potassium (mmol/L)	4.97 (4.5–5.48)	4.89 (4.5–5.47)	5.07 (4.47–5.48)	NS
Calcium (mmol/L)	2.12 (2.02–2.20)	2.14 (2.03–2.22)	2.12 (2.01–2.18)	NS
Magnesium (mmol/L)	0.86 (0.81–0.92)	0.86 (0.81–0.96)	0.84 (0.81–0.91)	NS
Phosphorus (mmol/L)	2.01 (1.64–2.28)	0.86 (0.81–0.96)	0.84 (0.81–0.91)	NS

*p*—comparison of children born at 30–34 weeks and 35–36 weeks; NS–non statistical.

**Table 3:** Examined parameters in premature neonates and the reference group—median values and interquartile range (IQR).

Parameters	Premature Neonates (Weeks) (*n*)			Reference Group	*p*_1_	*p*_2_	*p*_3_	*p*_4_
	(30–36) (60)	(30–34) (28)	(35–36) (32)	(≥37) (28)				
	Median (Q1–Q3)							
Urinary creatinine (mg/dL)	23.88 (3.43–43.58)	14.95 (9.47–25.05)	35.26 (21.38–57.73)	82.32 (38.99–118.66)	<0.01	<0.01	<0.01	<0.01
Serum creatinine (mg/dL)	0.66 (0.61–0.75)	0.64 (0.59–0.68)	0.70 (0.63–0.76)	0.68 (0.55–0.80)	NS	NS	NS	NS
eGFR (mL/min/1.73 m^2^)	24.58 (21.71–27.11)	24.38 (21.45–27.82)	24.86 (21.99–26.60)	37.62 (33.75–47.97)	<0.01	<0.01	<0.01	NS
Cathepsin B/creatinine (ng/mg Cr.)	3.41 (2.33–4.47)	4.00 (2.82–5.12)	3.07 (1.95–3.90)	2.51 (2.00–3.48)	<0.05	<0.01	NS	<0.05

*p*_1_—comparison of children born at 30–36 weeks and the reference group; *p*_2_—comparison of children born at 30–34 weeks and the reference group; *p*_3_—comparison of children born at 35–36 weeks and the reference group; *p*_4_—comparison of children born at 30–34 weeks and 35–36 weeks. Cr.—creatinine; eGFR—estimated glomerular filtration rate; NS—non statistical.
